# Concerns and potential improvements in end-of-life care from the perspectives of older patients and informal caregivers: a scoping review

**DOI:** 10.1186/s12877-021-02680-2

**Published:** 2021-12-20

**Authors:** Mina Motamedi, Caitlin Brandenburg, Mina Bakhit, Zoe A. Michaleff, Loai Albarqouni, Justin Clark, Meidelynn Ooi, Danial Bahudin, Danielle Ní Chróinín, Magnolia Cardona

**Affiliations:** 1 Australian Centre for Health Engagement Evidence and Values (ACHEEV), University of Wollongong, Wollongong, NSW Australia; 2 Allied Health Services, Gold Coast University Hospital, Southport, QLD Australia; 3 Institute for Evidence Based Healthcare (IEBH), Bond University, Robina, QLD Australia; 4 Faculty of Medicine, UNSW Sydney, Sydney, NSW Australia; 5 Faculty of Health Sciences and Medicine, Bond University, Robina, QLD Australia; 6 Department of Geriatric Medicine, Liverpool Hospital, Liverpool, NSW Australia; 7 South Western Sydney Clinical School, UNSW Sydney, Sydney, NSW Australia; 8 Bond EBP Professorial Unit, Gold Coast University Hospital, QLD Southport, Australia

**Keywords:** End-of-life care, Frail elderly, Informal caregivers, Patient-centered care, Quality of health care, Scoping review

## Abstract

**Background:**

Overtreatment in advanced age i.e. aggressive interventions that do not improve survival and are potentially harmful, can impair quality of care near the end of life (EOL). As healthcare provider perspectives on care quality may differ from that of service users, the aim of this study was to explore the views of older patients near EOL or their caregivers about the quality of health care at the EOL based on their lived experience, and to identify healthcare service improvements.

**Methods:**

Medline and backward citation searches were conducted for qualitative or quantitative studies reported on the views of patients and/or informal caregivers about EOL care quality. Thematic analysis was used to summarise qualitative data (primary analysis); narrative and tabulations were used to summarise quantitative data (secondary analysis).

**Results:**

Thirty articles met the inclusion criteria. Five main qualitative themes regarding quality care emerged: (1) Effective communication between clinicians and patients/caregivers; (2) Healthcare that values patient preferences and shared decision making; (3) Models of care that support quality of life and death with dignity; (4) Healthcare services that meet patient expectations; and (5) Support for informal caregivers in dealing with EOL challenges. The quantitative articles supported various aspects of the thematic framework.

**Conclusion:**

The findings of this study show that many of the issues highlighted by patients or bereaved relatives have persisted over the past two decades. There is an urgent need for comprehensive evaluation of care across the healthcare system and targeted redesign of existing EOL care pathways to ensure that care aligns with what patients and informal caregivers consider high-quality patient-centred care at the EOL.

**Supplementary Information:**

The online version contains supplementary material available at 10.1186/s12877-021-02680-2.

## Background

Older people (aged 60 years and above) in the terminal phase of chronic illness are often subjected to unnecessarily aggressive or unwanted medical or surgical procedures, which may not be beneficial, and which may in fact impair their quality of care and safety at the end of life (EOL) [1]. The views of clinicians on challenges to deliver optimal EOL care point towards a combination of lack of skill, health system’s inadequate environment, lack of time, a culture of medicalisation, organisational leadership, and legislation [2]. However, consultation on patient preferences or family acceptability of specific management approaches near the EOL is still not widespread [3, 4], despite recurrent recommendations for inclusion of consumer perspective in health service improvement proposed for over two decades [5]. Looking at the opinions of health service consumers—namely older patients and informal caregivers, including family members—individual studies indicate that issues of importance include symptom management, palliative care transition, engagement in decision-making, dying with dignity and place of death [6–8]. To our knowledge, no study has attempted to synthesise the views of consumers on what constitutes good quality care at the EOL.

In this scoping review, we focused on consumer perspectives to elucidate the broader domains and further characterise the concept of quality care, as delivered by health professionals in any setting, and beyond effectiveness of treatments [9], for older people dying of chronic illness. We focused on older people as they are frequent recipients of aggressive and potentially harmful treatments in the last few months of life [1]. The ultimate goal is to inform improvements in healthcare delivery for people near the EOL. This review is part of a larger initiative to examine consumer and clinician concordance and discordance and potential gaps in EOL care for older people (the protocol for entire project is available at https://osf.io/5u964/).

The study intended to respond to these research questions:

1. What are the perceptions of the consumer target group (patients, informal caregivers, families) about quality of EOL care services based on actual experience?
2. What are the identified areas for potential for improvements and suggestions based on these perceived gaps?

## Methods

### Search strategy

We checked protocols in PROSPERO and our information specialist (JCl) searched Medline from inception to June 2021 using the systematic review accelerator and polyglot search translator [10]. The full search strategy, inclusion/exclusion criteria, screening and data extraction processes are presented in Supplement 1, and Tables S1.1. and S1.2. English language only articles were identified, and title/abstract eligibility screening conducted independently by paired authors (MM, ZM, MC, MB, MO) with a third engaged in case of discrepancies. Manual checks of reference lists of eligible articles (backward citations) were also conducted by two authors (MM, MC).

### Inclusion and exclusion criteria

The population of interest was older patients (defined as 60+ years) near EOL (defined by authors of eligible articles as either terminal, incurable, dying, palliative, or EOL), and/or their relatives/informal caregivers. For articles that were potentially eligible but where EOL status was not explicitly stated, we used validated criteria for establishing EOL status based on an objective checklist (the CriSTAL tool) [11] at the full text assessment stage. If the mean or median patient age was 60+ years, at least 4 risk factors for death from the checklist needed to be present. If mean or median age of patients in the article was 80+ years then two risk factors sufficed to classify them as being at the EOL.

We adopted an inclusive approach, covering any setting where health professionals were the providers of any type of EOL care: hospitals, community services, primary care, hospice, or residential aged care. Our target outcomes were any opinions on in/appropriateness of care, experience, perception, views, and/or dis/satisfaction with healthcare quality—reported qualitatively or quantitatively—and heath service priorities, gaps, challenges, or suggestions for improvement. Those related to terminal care provided by family/informal carers were excluded. We included qualitative studies such as in-depth interviews, focus group discussions, Delphi studies, mixed methods, but also opinion/satisfaction surveys, research letters (if results presented), and conference abstracts. We excluded case studies, retrospective record reviews, studies of patient complaints or medical errors, studies including lay people who were not health service consumers, clinician perspectives, and protocol papers.

### Screening and data extraction

Paired reviewers with general practice and gerontology backgrounds (MM, MC, MO) independently screened all titles and abstracts using Rayyan software and involved a third general practitioner (MB) to discuss and resolve uncertainties or eligibility discordance. Full text eligibility was then conducted independently by pairs of reviewers (MM, MC, MB, ZM). For the qualitative studies a pre-designed template was used (MM) to extract the author, year, country, sample size, study type (focus group, in-depth interviews), setting, target group (patients, caregivers, both) and factors related to care quality including perceptions based on lived experiences. For qualitative studies, four authors (MM, ZM, MB, LA) extracted data and one (MM) developed the framework and mapped the articles. For the quantitative studies paired reviewers (MC, ZM, MB) screened full text and one (MC) extracted study characteristics (author, year, country, target group, setting and study objective/domain covered) and mapped the survey results using the framework developed for the qualitative analysis after all qualitative studies had been analysed. Another author (MB) reviewed and provided feedback on the presentation of survey results. At least one of the qualitative reviewers (MM/ZM/MB/LA) checked the contents and interpretation from both data extractions.

### Analysis

#### Qualitative studies: Primary analysis

To ensure methodological transparency and credibility, we used an iterative and reflective content analysis approach as recommended by Nowell et al [12]. Initial coding of semantic themes was completed by the authors who extracted the data (MM, ZM, MB, LA) using NVivo software to document themes, definitions, quotes and decisions, with the assistance of three other authors (MB, LA, CB). Development of themes, including both semantic and latent content, was refined by two authors (MM, CB) with input and discussion with all authors in regular meetings, including MC who is an EOL researcher. Final themes were developed by one author (CB) and checked against the original texts for reflexivity. Findings are presented as diagrams and tables with supporting quotes when available. DN provided clinical geriatrics expertise and contextual feedback. We did not undertake risk of bias assessment as the purpose was to identify opinions on care provided rather than to measure treatment quality.

#### Quantitative studies

Quantitative studies were investigated to identify if findings supported the qualitative conclusions, and whether they provided estimates of magnitude for the issues identified. Envisaging that studies would likely be heterogeneous in terms of methodology and target population, we planned to present survey results in tables, without any attempt to pool or meta-analyse numeric estimates.

## Results

We identified 17 studies published between 1997 and 2021 met the inclusion criteria. They were twelve fully qualitative designs and five thematically analysed the open-ended questions of surveys. The data derived from six countries (Australia, Canada, Germany, Norway, UK, and USA), involved 10,260 subjects. The flowchart of study selection is presented in Supplement 1, Fig S1.1.

In terms of study design, nine studies exclusively reported in-depth interviews [13–21], two combined focus groups with interviews [22, 23], one combined interviews with medical records data [24], and five conducted surveys with open-ended questions analysed using qualitative methods [25–29]. Studies included patients and/or relatives from single or combined settings in family homes (n= 6) [14–17, 23, 25], residential aged care (n=4) [16, 22, 24, 26], hospice (n=4) [13, 18, 19, 26] and hospital settings (n=3) [16, 24, 26].

**Fig 1.**
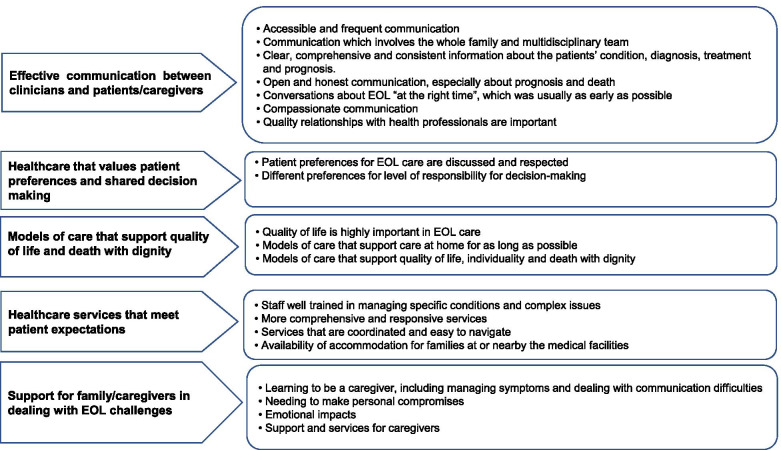
Themes and subthemes of quality EOL care emerging from the thematic analysis of the perspectives of patients and their family/caregiver

Five main themes emerged from the qualitative studies. Various aspects of receiving care at the EOL were discussed, such as expectations of quality of health care delivery, health care systems’ strength and weaknesses; and satisfactory services and their interactions with desired EOL care. Common identified themes, despite their overlapping issues, are categorised in five domains as illustrated in Supplement 2, Table S2.1 and Fig. 1:

### Theme 1: Effective communication between clinicians and patients/ caregivers

Across all included studies, communication was identified as a critical component in the provision of high-quality EOL care [13, 15–19, 22–26]. Patients and their family/caregivers valued communication which had a number of key features (Fig 1). A lack of good communication and relationships with health professionals led to a sense of abandonment and lack of trust in health professionals, which in some cases caused suspicion or confusion about the circumstances of treatment or death [16, 19, 26].

#### Accessible and frequent communication

Patients and their informal caregivers valued access to health professionals, including sit down discussions and the ability to contact clinicians for more information about diagnosis, prognosis and treatment, especially at times when their loved ones’ condition had changed [13–19, 22, 25, 26]. They felt communication should be frequent and ongoing in order to meet changing information needs, and that health professionals should be more responsive to attempts to contact them [16, 19, 20, 25, 29]. Family members also wanted to be able to have access to health professionals for a discussion after the death of the patient [25].

#### Communication that involves the whole family and multidisciplinary team

Opportunities for extended family (i.e. beyond the immediate family members) to have consistent information for clinicians’ coordinated communication [28] or to be involved in key discussions, e.g. family meetings, were appreciated [19, 25, 26]. It was also noted that input from the multidisciplinary team was important [13].

#### Clear, comprehensive, and consistent information about the patients’ condition, diagnosis, treatment, and prognosis.

Family/caregivers noted the need for clear, comprehensive, and consistent information about the patients’ condition/diagnosis, symptoms, treatment, prognosis/trajectory of the condition, and required nursing care [13–15, 17–19, 22, 25, 26]. In particular, consumers valued a clear diagnosis/prognosis, and information about options for management and available services [13, 25, 27]. While the preference was usually for more information, some caregivers appreciated the health professionals asking them how much they wanted to know [15, 20]. It was common for patients and family members to report receiving inadequate or contradictory information, particularly about the patient’s diagnosis and prognosis [13, 14, 16–19, 22, 25–28].

#### Open and honest communication, especially about prognosis and death

Patients and their family/caregivers frequently mentioned the need for clinicians to alert families about the patient’s final hours [28], and be open and honest about the patient’s prognosis, with a focus on ensuring the likelihood of death was communicated clearly, without using jargon, euphemisms like ‘not doing well’, or speaking in general terms like ‘needing hospice’ [13, 15–17, 19, 22, 23, 25, 26]. Family members felt that straightforward and realistic information from health professionals was more valuable than encouragement to keep up hope, which caused confusion [19, 26]. In particular, lack of clear understanding sometimes led to families pursuing undesirable curative treatments, or confusion about why the patient was receiving treatment if they were not going to recover [19, 26]. A clear understanding the patient was going to die allowed the family to appropriately plan and make decisions [26].

It was acknowledged that initiating conversation about EOL is difficult, especially when the patient is "not that sick”, and several factors need to be considered in the conversation [22, 25]. These included:

1. Cultural influences on how EOL and death are approached, and the fact that it is uncommon to talk about death in many cultures [23].
2. Patients and their family/caregivers’ confidence and ability to initiate or engage in EOL discussions, including issues of denial [13, 15, 17, 19, 22, 23].
3. Variability in the information patients and family are ready, willing and able to hear [15]. Some described not wanting to know all the information, for example that death was a possibility, or how long they were expected to live [15, 22, 26]. However; some family members who reported being resistant to hearing this information acknowledged in retrospect that it was necessary for them to know [26].

#### Conversations about EOL “at the right time”- usually as early as possible

Patients and their family/caregivers preferred that discussion about EOL care be initiated clearly by clinicians at “the right time” [22], which meant different things for different people. It was usually preferred to have an informative and comprehensive discussion about patient’s condition as early as possible, but it was found that EOL conversations were sometimes initiated when the patient's health had significantly deteriorated, which was seen to have negative consequences [13, 16, 19, 22, 23, 25, 26]. However, some patients and caregivers felt that conversations about EOL care should wait until the patient was closer to death, especially as they felt the patient’s opinions may change [22, 24]. Timeliness of communication was particularly important for the notification of imminent death, to enable loved ones to adjust plans to spend time with the patient and say goodbyes [16, 19, 25, 26]. Several studies mentioned that caregivers felt they were not given a sense of how soon death was likely [16, 25, 26]. And in some cases, prognostic uncertainty meant that the patient died earlier than predicted, causing distress for loved ones who thought they had more time [16, 19].

#### Compassionate communication

Many studies showed that patients and caregivers placed a lot of value on compassionate communication from staff. Compassionate communication was seen to be respectful, sensitive, sympathetic, and balanced the need for honesty with the need for hope [13–16, 19, 23, 25, 26]. Also emphasised was the need to feel cared about by health professionals, and to be recognised as individuals rather than numbers [19, 25]. In several studies, participants highlighted appreciation for nurses’ personal qualities, like flexibility [27], generosity, compassion, empathy [29] and sense of humour, far more than technical skill [14]. Some reported receiving difficult news in a way that was perceived to be abrupt and blunt which caused distress and made the news more difficult to process [19]. It was also important to family members to have health services recognise the death of their loved one in some way [25].

#### Quality relationships with health professionals are important

Quality of relationships with health professionals, especially collaboration, respect and trust were seen as important for communication and quality care [13, 14, 16, 17, 19, 23]. Family members expressed a preference for hearing information about a prognosis of death from a health professional they had a rapport with, rather than a ‘stranger’, and having a personal as well as a professional connection with health professionals [13, 14, 16, 17, 19].

### Theme 2: Healthcare that values patient preferences and shared decision-making

Strongly related to the theme of communication, patients and family spoke about care which involved discussions of patient preferences and shared decision-making, an inclusive modality of clinical practice where patients receive information on treatment options, harms and benefits, and are supported by clinicians in jointly deciding on the course of action [30]. Decision-making relied heavily on good communication in which they had a realistic view of the prognosis [19] and a good understanding of the management options available [13, 17].

#### Patient preferences for EOL care are discussed and respected

In some cases, families felt overlooked and wanted to be listened to as they knew patients better than the clinical team [21]; and in most cases patients wanted to be respected as an individual with their own personal values and preferences for care, and discuss these with healthcare professionals and family [13, 15, 22, 23, 25], while a minority of patients felt a specific discussion was not required [22]. In many cases, a discussion had not taken place even through it was seen as important that preferences were known [15, 22]. This may have been because the discussions were left to chance, or it was seen as the patients’ role to initiate the discussion [22].

Caregiver opinions on advance care directives were mixed. Some caregivers felt they were not needed as they felt they knew their loved ones’ wishes, even if they had not had a specific conversation [13, 22]. In other cases, family/caregivers saw adherence to written directives as essential [23].

Many barriers or complicating factors to these discussions were emphasised. Discussions about preferences for EOL care are emotionally difficult, patients’ cognitive deterioration can affect their participation in these discussions, and poor communication from health professionals might mean they do not have a full understanding of the options [13, 17, 22, 23]. Another complicating factor was that in some cases patients and family withheld negative information from one another, hoping to protect the other party [13, 15, 17]. Preferences may also change over time, especially towards the EOL as patients may be less likely to pursue life prolonging treatment [15, 22].

In a few cases patients and caregivers disagreed on the best management option [15, 17, 24]. In many examples patients’ expressed wishes were not upheld [15, 17, 23, 24]. For instance, some caregivers accepted the patient’s wishes reluctantly, while others gave medical orders against patient’s wishes after losing their capacity to make decisions [15, 17, 24]. And in two studies, family members reported that health professionals did not follow patient’s request to stop treatments [28], or adhere to advance care directives, with the reasons for this unclear to them [23].

#### Different preferences for level of involvement in decision-making

While being included in decision-making conversations was important for most, there were variations in the level of responsibility patients and their family/caregivers wanted in making final decisions. Some patients preferred to leave all medical decisions to the staff, others wanted to be involved in the decision-making but trusted staff to make final decisions and, least commonly, some wanted to be solely responsible to make decisions [22]. Some patients and family were not concerned whether they had control over specific decisions [16].

Generally, family members wanted to be involved in decision-making if the patient lacked capacity, but they did not want the burden of authority [22]. Some even expressed fear about being responsible for decisions about care, and relief that clinicians led decisions or that the patient had expressed clear directions [15, 22]. Mixed responses to urgent decision-making were observed. Some caregivers preferred the medical staff making urgent decisions and informing the family afterwards, while others wanted to be informed prior to any decisions being made if at all possible [15, 22, 25].

### Theme 3: Models of care that support quality of life and death with dignity

Both patients and family/caregivers spoke about the importance of quality of life and death with dignity in EOL care, and wanted models of care that supported this. Models of care refer to the way in which health services are delivered in terms of service goals, modality, accessibility, and location,for example care at home.

#### Quality of life is highly important in EOL care

Quality of life was emphasised as the most important goal in EOL care, often above prolonging existence [16, 17, 22, 23]. Quality of life had many meanings, including elements of autonomy, independence, achieving goals, being comfortable, lack of pain, being able to do things a person enjoys and maintaining sense of self through valued activities [14, 23, 24]. However, patients acknowledged that their standard of 'good quality of life' may change over time [23].

#### Models of care that support care at home for as long as possible

Many studies outlined that patients and family/caregivers preferred to receive care at home for as long as possible and spend time with their loved ones [14, 16, 17, 23, 25, 29]. This did not necessarily mean dying at home, as some caregivers were resistant to this idea [17]. Patients and their family/caregivers felt that eliminating administrative barriers to accessing outpatient and community services would constitute better EOL care models to support patients who receive care at home [23, 25]. Some were very grateful for staff treating patients as a member of their family [27].

#### Models of care that support quality of life, individuality, and death with dignity

Some patients reported that the system of care did not take into account quality of life and individual wants/needs, as needing to be on someone else’s schedule diminished autonomy [14, 23]. Death with dignity was also important, including a private room for death [21], and allowing loved ones to spend time with the body at home if possible and desired [25].

### Theme 4: Healthcare services that meet patient expectations

Patients, family and caregivers also had other expectations of the quality of EOL care, such as highly skilled staff; more comprehensive and responsive services; and coordinated and easy-to-navigate services. Some patients balanced this by emphasising realistic expectations of care and accepting that care providers are human and not perfect [14, 16].

#### Staff well trained in managing specific conditions and complex issues

Patients and caregivers felt staff could be better trained in dealing with older patients, patients with specific conditions like dementia, and in managing complex issues like pain, challenging behaviours and psychosocial issues [16, 24, 25].

#### More comprehensive and responsive services

Requests for improved comprehensiveness and coverage of care services, including hospital, nursing home, outpatient and out of hours services were also made [17, 25]. In particular, caregivers felt there was too much expectation of them to help with care in inpatient settings [16, 25]. Some family members felt that staff were inattentive and rushed as a result of understaffing, and felt that they had to be there or the patient would not get good care [16, 25]. Caregivers also wanted services that were more responsive, for example to escalation in symptom management, or whole family needs [17, 19].

#### Services that are coordinated and easy to navigate

Family members often found services difficult to navigate, and would value better coordination and continuity of care [17, 18, 23]. Key issues were liaising with multiple service providers, multiple visits, conflicting prescriptions, non-local services, mobility issues, cost of transport, financial and insurance barriers [16, 17, 23, 25].

#### Availability of accommodation near health facilities

There were different experiences about the ability of families to stay overnight near the dying person’s hospital. Two studies showed that staying near the dying person’s hospital was important and reflected good quality of care. While some families appreciated social workers and other staff making arrangements for overnight stays near the hospital [27, 28], while others expressed disappointment that they did not have opportunity for proper final goodbyes due to lack of an adjacent hotel [27].

### Theme 5: Support for family/caregivers in dealing with EOL challenges

Family members and caregivers also spoke about the challenges facing them, asking that health services be sensitive to this, and provide supports where possible.

#### Learning to be a caregiver, including managing symptoms and dealing with communication difficulties

Families talked about how challenging it was to learn to be a caregiver and manage symptoms, particularly pain [17, 20, 23, 25]. They found managing symptoms to be more challenging when compounded by language, cognition, behaviour, or emotional lability issues that hindered communication; creativity in communication methods and familiarity with the person was reported to improve this [18]. Variability in the patient’s condition and responsiveness to strategies from day to day also compounded the difficulty in dealing with symptoms [16, 18, 19].

#### Needing to make personal compromises

Family/ caregivers reported making significant financial, career and personal compromises when a family member was at the EOL, and putting their own lives ‘on hold’ [23]. Care at the EOL also puts a strain on other relationships, for example cases where the burden of care was uneven between family members resulting in disagreements, or when time with other family members including young children was more limited [23].

#### Emotional impacts

Emotions reported by informal caregivers while providing EOL care included feelings of guilt, denial, distress, confusion, sadness, mental exhaustion, resignation, and conflicted feelings about decisions to no longer prolong life [17–19, 23, 25, 26]. On the other hand, gratitude for clinicians’ supportive expressions of condolences, understanding and enablement of longer time to stay with their loved one in the aftermath were also observed [28].

#### Support and services for caregivers

Given the challenges to providing EOL care, caregivers requested support specific to their emotional, spiritual, psychosocial and practical needs [18, 23, 25]. Suggestions ranged from health professionals simply recognising the caregivers’ role and inquiring if they needed help, to connection with more formal alternative support such as respite services [18, 25] including earlier palliative care referral [27]. Caregivers felt they needed proactive support and education in providing care, rather than input at the point where symptoms became unmanageable [18, 23, 24]. Finally, caregivers and patients’ family also mentioned the need for sympathetic support, bereavement care, and connection to grief support services after the patient had died [16, 25].

Exemplary quotes for all the above themes are in Supplement 1, Table S1.3.

#### Findings from the quantitative studies

For the quantitative component, thirteen quantitative studies published between 2003 and 2019 were also eligible for inclusion: two nationwide surveys of bereaved relatives in Japan [31, 32], three national surveys in USA [33–35], three surveys of bereaved relatives [36–38], and five surveys of chronically ill terminal patients or their families [39–43]. Response rates ranged 27%-100% and mean age across studies ranged from 60-86 years. Perspectives from 11,626 participants from four countries (Canada, USA, Japan, The Netherlands) covered satisfaction with support received, elements of EOL care, and opportunities for improvement. Tables S3.1 and S3.2 in Supplement 3 give the details of individual study objectives, response rates and domains covered in each of the 13 surveys. While they did not all fit perfectly into the above subthemes, generally these findings supported various aspects of the framework derived from the thematic analysis (Summary 3.1 in Supplement 3).

## Discussion

Through our review of 23 qualitative research articles, we were able to synthesise older patients’ and informal caregivers’ views on EOL care quality into 5 main themes that were supported by the data from quantitative studies: (1) Effective communication between clinicians and patients/caregivers; (2) Healthcare that values patient preferences and shared decision-making; (3) Models of care that support quality of life and death with dignity; (4) Healthcare delivery that meets patient expectations; and (5) Support for family/caregivers in dealing with EOL challenges

The findings of this review highlight sub-optimal communication to be a major and persisting issue over the past two decades, impacting on patient-clinician relationships, trust, and quality of care at the EOL, especially in more recent times. Older patients and relatives often raised clear communication about prognosis and treatment effectiveness as major needs towards EOL [44]. Bereaved families of patients with dementia would have liked healthcare professionals to spend more time with them to help manage multiple burdensome symptoms, and to have received more emotional support during the terminal phase and in the aftermath [25]. Supporting this to occur in practice is a challenge, as good quality patient-clinician communication can take time and increase staff workloads. However, the fact that this remains a strong theme in the literature points towards the need for strategies to further build this capacity into the role of clinicians by remunerating additional time [45, 46] to optimise the provision of quality EOL care.

Another perceived gap in quality care at the EOL was the lack of systematic integration of patient choice in treatment decision-making. Identified contributors to this included cognitive impairment and conflicting family-member views regarding level of responsibility and content of advance health directives. A common source of dissatisfaction was the loss of autonomy or dignity towards the end, this ranging from reduced EOL quality due to unnecessarily prolonged treatments, through unmet needs relating to place of death, to lack of privacy in the aftermath. The complexity of navigating the health system was also highlighted, including suboptimal responsiveness from healthcare staff and expectations that informal caregivers will supplement the loved ones’ care delivery in both hospitals and residential aged care facilities. This transfer of responsibility was also a contributor to the caregiver burden and limited emotional and technical support experienced when patients were managed at home. A recent qualitative study also found that patients and caregivers welcome services that meet personalised supportive care needs, and provide streamlined logistics to navigate the health system (our theme 3) and offers support for families (our theme 5 )[47].

Quality of dying can be conceptualised as alignment with preferred place of death, symptom control, and satisfaction with communication with/from the managing health professionals [48]. Respect in clinician-family communication, interpreters across cultures, spiritual support and facilities conducive to family gathering are also aspects considered important in good EOL care by bereaved relatives [49]. The consumer expectation of communication and ability of the health system to meet those needs has been reported to differ from the clinicians’ perspective [50]. It is clear that service features and solutions have been proposed for some time, and many models of care are in place, but it appears that implementation of the care quality components remains unsatisfactory for consumers.

The repeatedly-emerging expectation of respect, sensitivity, compassion and empowering communication from healthcare professionals found in our review as integral to ‘good quality care’ has been previously reported [51]. Perception of deficiencies in information received- about both the incurable or imminent deadly nature of the illness-, choices for place of death and availability of palliative support for non-cancer conditions have also been reported in other studies not eligible for this review [52, 53]. Three recent studies pertaining to death in hospice [54] or hospital [55] also highlighted clinician team communication ‘failures’ as the main factor influencing their perception of (poor) quality of care.

Our findings from an older population (60+ years) resemble those from other studies of younger age groups. A recent survey of 356 relatives of deceased adults (aged 18 to 80+ years) also identified communication, privacy and emotional and spiritual support as areas that required improvement in acute hospital’s end-of-life care [56]. Likewise a survey of 434 critically ill patients and 176 younger relatives (mean aged 56.5 years (SD 13.9) about their perceived priorities indicated that honest communications, being treated with dignity, respect for values an preferences, dying in their preferred location and spiritual support were some of the important elements of care quality [41]. We had anticipated that dementia and surrogate decision-making for the cognitively impaired would have featured more prominently in our older target group, but this did not emerge as a priority issue for consumers. This strongly suggests that a common desire for hospitalised dying patients and families regardless of age, is to have control over the information received, avoid suffering, receive support to die at home, and have opportunity to share time with family. This collectively understanding of care quality may facilitate implementation of policies and practices across services and patient groups.

### Potential solutions

The results of this scoping review identified several areas that may be targets for future interventions to improve EOL care. They included honest communication, greater availability of clinicians to clarify issues, shared decision-making [42], and simulated consultations [57]. Evaluation of their success in real-life practice is warranted. Formal care plans documenting patient’s wishes and treatment preferences for future critical illness have also been proposed as potentially reducing the delivery of non-goal-aligned treatments [58]. Future EOL care planning post-discharge has proven effective in reducing unnecessary hospital returns, without impacting adversely on anxiety, distress or quality of life [59]. However, the willingness of holding and EOL discussion is known to vary. The likelihood is higher for people who have played a caregiving role of a dying loved one in the past, and lower for males, younger consumers and those who do not have a family doctor [60]. Uptake of documented care plans may be hindered by lack of availability of care plans directives, ‘out of date’ plans, suboptimal documentation (wishes not clear enough and/or subject to misinterpretation), instructions which are not relevant to the current clinical situation, or families opposing execution of the care plan [61, 62]. To overcome this, another suggestion has been the use of reliable and validated instruments to more clearly elicit patient values in the face of serious illness and link them to potential treatment alternatives in the context of shared decision-making [63]. Yet confidence in sensitive communications and identification of patients’ readiness for the conversation requires training. While there are skills courses [64] and communication guidelines available [65], evaluation of their effectiveness is scarce, or interventions are of low quality and/or failed to reduce overtreatment near the EOL [66], or outcomes are subjective and self-reported [67]. Public education on advance care planning and engagement in normalisation of the discussion led by civic leaders and community-based organisations has also been recommended [68].

Variation in consumer readiness to talk about death is not unusual. Some patients and caregivers are known to proactively initiate discussions and documentation; they welcome more opportunity for active participation in decision-making, and may request lay-terms education and written information on their illness trajectories and management [69]. Other individuals, for cultural or other reasons, may adopt denial, preferring non-disclosure to distressing prognostic information [70]. There is no consensus on the ethical response to patient preferences ‘not to know’. Earlier initiation of advance care planning discussions by clinicians is hypothesised to contribute to the solution, but proactive uptake is not high [71]. A recent randomised trial in primary care found the use of conversation starters in group visits helpful in increasing readiness for advance care planning [72]. Likewise a trial of a brief nurse intervention for older patients, using visual materials on goals of care preference, led to improved knowledge and patient readiness to discuss options, and a greater proportion choosing not to undergo cardiopulmonary resuscitation [73]. Another enhancer of patient satisfaction with EOL care is earlier referral to palliative care consultation. A retrospective study [74] and a prospective pilot outreach outpatient close to the patient’s home have shown reduction in aggressive healthcare interventions and improved quality EOL experience [75]. While these studies are encouraging, higher-level evidence is required.

Several health system issues have previously been identified as impairing quality palliative care [76], such as clinician lack of familiarity with the process, lack of continuity of care with a sequence of professionals providing assessment, and lack of consumer confidence in or awareness of their right to ask questions. These can potentially be addressed through institution-specific orientation of clinicians, and refresher or update courses for changing procedures and evolving work environments. Furthermore, while informal care by relatives, friends and other community groups are provided and welcome near the EOL [77], the consumers in this review warned of the caregiver burden, possibly under-appreciated by healthcare professionals, and highlight the need to provide holistic care that encompasses the carer as well as the patient.

### Strengths and limitations

This scoping review combined qualitative and quantitative articles published in English over two decades and up to 2021 in several health systems to characterise the consumer perspective on expectations of what good quality of care at the EOL should incorporate. Rigorous systematic review methods were used in this review with paired authors involved in the screening and selection process. There are some limitations to this study. Firstly, searches were only conducted in one database, Medline. However, Medline is one of the largest biomedical databases indexing over 26 million articles. The electronic database search was supplemented with backward citation searches. Secondly, as this is a scoping review, no risk of bias assessment was conducted. Finally, as within any review, we can only comment on the available data, and acknowledge limitations of the eligible studies, including issues such as potential for exclusion of some countries if studies were not published in English language, selection bias amongst participants, and unknown representativeness.

### Implications for practice

It is disheartening to note that many of the issues identified in the 1990s publications were still mentioned in the articles released in the 2010s, and crossover of unmet needs was reported by older patients and consumers of all ages. This suggests that despite recurrent evidence highlighting gaps, we have failed to bridge these. Whatever advances we may think we have made as regards education regarding EOL care and the need for shared decision-making, incorporation of communication into healthcare professional training, and investments in palliative care services, these have not yet translated into concrete improvements in EOL care, at least as supported by the available data. A multifaceted, multidisciplinary approach is likely to be needed, which spans public health, primary care, community care, acute healthcare and residential care, focussing on key points that will aid the journey towards the EOL: normalising discussions about death, advance care planning, acknowledging frailty and accumulating morbidity, recognising that prognosis is dynamic, upskilling and empowering clinicians to provide sensitive, appropriate, patient-centred care, integrating care and improving communication across and between the various healthcare sectors, matching resources to patient (and population) needs, and embedding patients and families at the heart of decision-making. Furthermore, where interventions are shown to be promising in small-scale studies, these need to be evaluated first in larger populations before translating it into everyday practices/guidelines, and efficacy in real-world setting assessed. Interventions need to be adapted for local context, in order to meet the changing needs of patients and caregivers [78].

### Areas for further research

Overall, the evidence for effective strategies to enhance quality of care that meets patient and family needs is low and results need to be viewed with caution. Areas of difficulty in decision-making have been highlighted for surrogates of patients dying of cancer [79]; future research can expand this to the most difficult decisions to be supported by health professionals treating other life-threatening, chronic, non-cancer conditions. Important evidence gaps still remain on effective strategies to prevent medical professionals or family overriding the patient’s expressed wishes. In light of the widespread patient preference to die at home, evaluations of the emotional and financial impact of home deaths are warranted, as at present, community-based resources are largely insufficient to support patients without burdening informal caregivers. Ultimately, translation of evidence into practice and adaptation to local context is needed to help provide optimal quality of care at the EOL.

## Conclusions

This scoping review identified five themes that reflect older patients and informal caregiver perceptions of quality care at EOL. Many of the issues highlighted by patients or bereaved relatives are not new and have persisted over two decades, highlighting the need to embrace a multifaceted, multidisciplinary approach that addresses the many levels of the EOL experience, and ensuring that promising initiatives are translated, evaluated and scaled up. These findings are relevant to clinicians, managers and policy makers and highlight a number of solutions that can implemented at every level to improve the quality of care at EOL. However, phase 3 trials are yet to demonstrate sustainable effectiveness of some of the proposed solutions at larger scale; and there is an urgent need for comprehensive evaluation of care across the healthcare system and targeted redesign of existing EOL care pathways to ensure care aligns with what patients and informal carers consider high-quality patient-centred care at the EOL.

## Supplementary Information

**Additional file 1.**

## Data Availability

All data for this review has been presented in tables, figures and supplementary materials. The authors can be contacted by email for further queries.
