# Supplementary material for: Concerns and potential improvements in end-of-life care from the perspectives of older patients and informal caregivers: a scoping review
Source: BMC Geriatr. 2021 Dec 20;21:729. doi: 10.1186/s12877-021-02680-2 (PMC8690959; doi:10.1186/s12877-021-02680-2)
Supplement: Supplementary file 1 — Additional file 1. [file 12877_2021_2680_MOESM1_ESM.docx]

**SUPPLEMENTARY FILES 1-3**

**Concerns and potential improvements in end-of-life care from the perspectives of older patients and informal caregivers. A systematic narrative review**

| **Supplement 1** | Inclusion/exclusion criteria, screening & data extraction |
| --- | --- |
| Table S1.1 | Full search strategy |
| Table S1.2 | Criteria to deem research subjects as being near the end of life (CriSTAL tool) |
| Figure S1.1 | PRISMA diagram: study selection and reasons for exclusion |
| Table S1.3 | Selected quotes to illustrate emerging themes and sub-themes from qualitative analysis of eligible articles |
| **Supplement 2** |  |
| Table S2.1 | Representation of domain consumer perceptions of quality of care near the end of life (EOL) |
| **Supplement 3** |  |
| Table S3.1 | Characteristics and context of quantitative studies by target population (N=13) |
| Table S3.2 | Consumer perspectives on experiences and care quality near end of life (Quantitative studies N=13) by target group |
| Summary 3.1 | Detailed findings from the quantitative studies (13 surveys) |

**Supplement 1. Inclusion/exclusion criteria, screening & data extraction**

***Inclusion and exclusion criteria***

The population of interest was older patients (defined as 60+ years) near EOL (defined by authors of eligible articles as either terminal, incurable, dying, palliative, or end-of-life), and/or their relatives/informal caregivers. For articles that were potentially eligible but where EOL status was not explicitly stated, we used validated criteria for establishing end-of-life status based on an objective checklist (the CriSTAL tool) [1] at the full text assessment stage. If the mean or median patient age was 60+ years, at least 4 risk factors for death from the checklist needed to be present. If mean or median age of patients in the article was 80+ years then two risk factors sufficed to classify them at EOL.

We adopted an inclusive approach, covering any setting where health professionals were the providers of any type of end-of-life care: hospitals, community services, primary care, hospice, or residential aged care. Our target outcomes were any opinions on in/appropriateness of care, experience, perception, views, and/or dis/satisfaction with healthcare quality- reported qualitatively or quantitatively- and heath service priorities, gaps, challenges, or suggestions for improvement. Those related to terminal care provided by family/informal carers were excluded. We included qualitative studies such as in-depth interviews, focus group discussions, Delphi studies, mixed methods, but also opinion/satisfaction surveys, research letters (if results presented), and conference abstracts. We excluded case studies, retrospective record reviews, studies of patient complaints or medical errors, studies including lay people who are not health service consumers, clinician perspectives, and protocol papers.

***Screening and data extraction***

Paired reviewers with general practice and gerontology backgrounds (MM, MC, MO) independently screened all titles and abstracts using Rayyan software and involved a third general practitioner (MB) to discuss and resolve uncertainties or eligibility discordance. Full text eligibility was then conducted independently by pairs of reviewers (MM, MC, MB, ZM). For the qualitative studies a pre-designed template was used (MM) to extract the author, year, country, sample size, study type (focus group, in-depth interviews), setting, target group (patients, caregivers, both) and factors related to care quality including perceptions based on lived experiences. For qualitative studies four authors (MM, ZM, MB, LA) extracted data and one (MM) developed the framework and mapped the articles. For the quantitative studies paired reviewers (MC, ZM, MB) screened full text and one (MC) extracted study characteristics (author, year, country, target group, setting and study objective/domain covered) and mapped the survey results using the framework developed for the qualitative analysis after all qualitative studies had been analysed. Another author (MB) reviewed and provided feedback on the presentation of survey results. At least one of the qualitative reviewers (MM/ZM/MB/LA) checked the contents and interpretation from both data extractions.

**Supplement 1. Table S1.1 Full search strategy**

| **Key articles to guide search** |
| --- |
| 30446268[uid] OR 27846832[uid] OR 30868725[uid] OR 30614161[uid] OR 26124510[uid] OR 24644204[uid] OR 22555775[uid] OR 26598036[uid] |
| **Search terms** |
| (Aged[Mesh] OR Elderly[tiab] OR Older[tiab] OR decision-makers[tiab] OR Surrogates[tiab] OR Patients[tiab] OR Carers[tiab])  AND  (“Terminal Care”[Mesh] OR "Palliative Care"[Mesh] OR “Frail Elderly”[Mesh] OR “End of life”[tiab] OR “Last year of life”[tiab] OR “Limited life”[tiab] OR Frail[tiab] OR Death[tiab] OR Dying[tiab] OR Palliative[tiab] OR “Intensive care”[tiab])  AND  ("Patient Preference"[Mesh] OR Perception[tiab] OR perspective[tiab] OR perceived[tiab] OR experience[tiab] OR Experiences[tiab] OR opinion[tiab] OR views[tiab] OR satisfaction[tiab] OR discordance[tiab] OR discrepancy[tiab] OR dissonance[tiab])  AND  (Consultation[tiab] OR Communication[tiab] OR “focus groups”[tiab] OR “Focus group”[tiab])  AND  ("Surveys and Questionnaires"[Mesh] OR "Interviews as Topic"[Mesh] OR Qualitative[tiab] OR Surveys[tiab] OR questionnaires[tiab] OR Interview[tiab] OR Interviews[tiab] OR Survey[tiab] OR “Thematic analysis”[tiab]) |

**Supplement 1. Table S1.2 Criteria to deem research subjects as being near the end of life (CriSTAL* tool)**

|  | **Sex 1. □ Male 2. □ Female Date of birth_____/_____/_____________** | |
| --- | --- | --- |
| **☐**  **☐** | **Age >65** *(1 point)*  Eligible for admission via emergency Department or at least 1 night in the ED *(1 point)* | |
| **☐** | Nursing home resident / or in supported accommodation *(either: max 1 point)* | |
| **☐** | **Clinical Frailty Score >= 5** ☐ Yes *(1 point if Yes)* ☐ No Actual CFS score *____* | |
| **☐** | **Meets 2 or more** selected deterioration criteria on admission *(max 1 point if it meets >2 RRT criteria)* | |
|  | **☐** | 1-Decreased LOC: Glasgow Coma Score change >2 or AVPU =P or =U |
|  | **☐** | 2-Respiratory rate <5 or >30 or Oxygen Saturation <90% |
|  | **☐** | 3-Hypoglycaemia: BGL ­1.0 - 4.0 mmol/L |
|  | **☐** | 4-Repeat or prolonged seizures (single episode >5 minutes or >2 episodes 24 hrs) |
|  | **☐** | 5-Low urinary output (<15 ml/hour or <0.5 ml/kg/hour) |
| **AND** | OTHER RISK FACTORS /PREDICTORS (*tick as many as relevant- max 7 points*) | |
| **☐**  **☐**  **☐**  **☐**  **☐**  **☐**  **☐** | **Personal history of active disease:**  1-Advanced malignancy  2-Chronic kidney disease  3-Chronic heart failure  4-Chronic obstructive pulmonary disease  5-New cerebrovascular disease  6-History of Or New myocardial infarction  7-Moderate/severe liver disease | |
| **☐** | Evidence of cognitive impairment *(tick as many as relevant – only 1 point if >1 mental condition)*  ☐Dementia ☐ Long term mental disorder  ☐ Behavioural Alterations ☐ Mental disability from stroke | |
| **☐** | Proteinuria on a spot urine sample: ++ or >30 mg albumin/g creatinine  ☐ Yes *(1 point)* ☐ No ☐ Don’t know | |
| **☐** | Abnormal ECG (atrial fibrillation, ventricular tachycardia, other abnormal rhythm or >5 ectopics/min, changes to Q or ST waves) *(tick as many as relevant – only 1 point if >1 abnormality)*  **☐** Yes ☐ No abnormality ☐ Don’t know | |
| **☐** | **Previous hospitalisation for at least one night in past year** (*only 1 point if >1 hospital admission)*  ☐ Yes  ☐ No ☐ Not documented | |
| **☐** | **ICU admission at previous hospitalisation in the past year** (*only 1 point if >1 ICU admission)*  ☐ Yes  ☐ No ICU admission at all **□** Unknown | |
| **☐**  **☐** | **Fall in the past 3 months** *(1 point)*    **Polypharmacy (6 or more medications) before admission** *(1 point if >6 medications)* | |

***Cri**teria for **S**creening and **T**riaging to **A**ppropriate a**L**ternative care

**Supplement 1, Figure S1.1 PRISMA diagram. Study selection and reasons for exclusion**


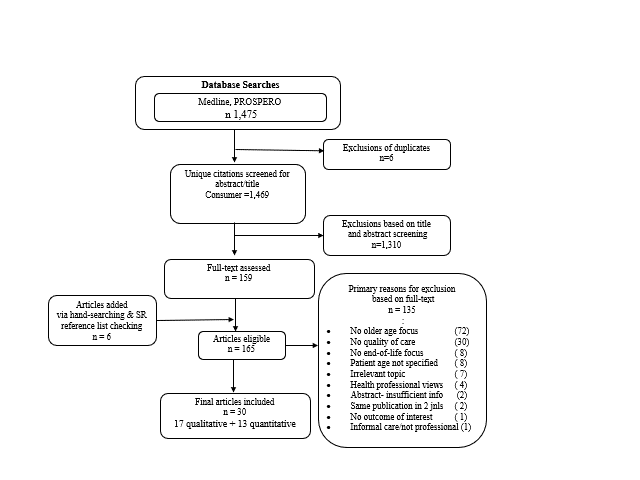


**Supplement 1, Table S1.3 Selected quotes to illustrate emerging themes and sub-themes from qualitative analysis of eligible articles (n=17)**

| **Theme** | **Subtheme** | **Study reference** | **Quotes from the article** |
| --- | --- | --- | --- |
| **Communication** | Accessible and frequent communication | Waldrop, 2012 | Sparse communication was defined as inadequate depth and frequency of contact with providers. Sparse communication occurred when caregivers tried repeatedly to get a provider to return calls or struggled to get answers to the questions they had about their loved one’s diagnosis, prognosis, treatment, and needs.  *The doctors at the hospital. and you could never catch a doctor. So now the doctor wouldn’t return calls. You can never catch a doctor on duty at the hospital. You could talk to the nurses but they can’t tell you anything. So for about a week I was going absolutely crazy yelling at my sister, ‘‘You’ve got to get in touch with this doctor.’’ She said, ‘‘They just won’t call me back.’’* |
|  |  | Armstrong 2020 | Poorly coordinated care across the healthcare providers  *Once they figured out what to do, they couldn’t execute it across the team, you know, so it’s so*  *many people. The hospice people take over for the doctor, and then the hospice people are*  *communicating with the in-house people, but the in-house people are in so many different shifts,*  *and—I’d call hospice. Hospice—says, “No. Your doctor has to write an order.”* |
|  |  | Muders, 2015 | Care and sympathy after their loved one’s death was another important concern for respondents. After bereavement, they mainly wished for talks or just a sign of sympathy from professionals:  *[I would have appreciated] to have a conversation with [the professionals] following the passing.* |
|  |  | Morris, 2020 | *“(They) said to call anytime and they could be reached. They listened to me when I made suggestions”* |
|  | Communication that involves the whole family and multidisciplinary team | Waldrop, 2012 | Responsiveness to the whole family’s needs was illustrated by the use of a family meeting to discuss looming decisions and increase comprehensibility. |
|  |  | Csikai 2010 | The caregivers in this study strongly suggested that professionals work together to provide information needed to make end-of-life care decisions. |
|  |  | Gray 2020 | Improved care team collaboration  *I believe your computer system needs to be better. Every time my dad was moved, and a new doctor came on, that doctor never knew what was going on. Either the record was not written into his case or doctor did not look at history.* |
|  | Clear, comprehensive, and consistent information about the patients’ condition, diagnosis, treatment and prognosis. | Waldrop, 2012 | Contradictory communication occurred when caregivers received variant explanations, descriptions, or prognoses from providers. Participants had difficulty comprehending the true nature of the situation given diffuse or vague language. In addition, participants described hearing different slants or spins to a message.  *I think that the doctors should have been more honest with us and let us know and they didn’t. They should have told us that they weren’t going to operate. They kept saying tomorrow. I used to get there by 9:00 and the doctor would be gone before I’d get there. And he would tell our son, she’s not in good shape now. But that’s the only time I guess I heard something. I never heard anything out of him. I never saw him in there.* |
|  |  | Agha 2021 | *‘‘The biggest issue in my opinion was that there were too many people (doctors) that were involved in taking care of him. I would ask a question of one individual and given an answer. One hour later, ask a question and given a totally different answer by someone else.’’* |
|  |  | Armstrong 2020 | Desire for better staff communication with family members  *The other things, um, I would’ve liked to see in these are, in the grand scheme of things, quite*  *minor. But the communication between the facility and the families of people in memory care*  *about such things as staffing changes was - suboptimal.* |
|  |  | Gray 2020 | While my father was in ________ VA, the doctor/nurses should have discussed my father’s prognosis. While he was being cared for I was never made aware of his condition of stage 4 cancer and dementia. It was explained to me that his care was for his ulcer wound, but they put him in a hospice room. The day he was discharged it was made clear that he needed to be in hospice, but I didn’t understand why they were telling me my father needed to be in hospice. |
|  |  | Agha 2021 | ‘‘The physician…was excellent! He communicated with us every day, updating us on his condition and helping us to make difficult EOL decisions.’’ |
|  | Open and honest communication, especially about prognosis and death | Fried 2008 | caregivers were pleased with the prognostic information they and their loved ones received.  open communication was seen as having many benefits, including preventing dissention among different family members and providing closure |
|  |  | Waldrop, 2012 | Participants described encounters with providers who delivered a poor prognosis or the news that a loved one was dying imminently. Participants described this kind of communication as blunt, short, abrupt, and direct.  *He just straightforwardly said, ‘‘She has cancer in a lot of her stomach, and it’s gone to her liver. I’m going to be honest with you, she will not be here for the holidays. I can’t give you how long, but it’s not going to be very long.’’ That was a shocker.* |
|  |  | Gray 2020 | Alerting family of patient’s final hours  *My husband was dead, and no one called me. His daughter went to see him and found him dead. They had to find the decedent clerk to come up for him.* |
|  | Conversations about EOL “at the right time”, which was usually as early as possible | Gjerberg 2015 | Irrespective of the experience with this type of conversation, who had taken the initiative or what had given rise to it, most relatives expressed that they wanted a conversation about the patient’s wants and preferences for end-of-life care, even when such conversations might be emotionally difficult. However, they would appreciate that the staff took the initiative, but stressed that it should take place at “the right time”. |
|  | Compassionate communication | Hanson, 1997 | most praised the skills of compassionate and attentive nurses.  *“ , . . beautiful balance between people caring about their patients and being medically top notch.”* |
|  |  | Morris, 2020 | the clinical team showed or communicated empathy, caring and concern. |
|  |  | Agha 2021 | *‘‘He wanted to come home and see his dog Molly. But he was unable to, so they allowed me to bring Molly to him. It put a smile on his face. The first in several months. Thank you for that.’’* |
|  | Quality relationships with health professionals are important | Hanson, 1997 | Families praised physicians who were *“. . . open, communicative, and collaborative.”* |
| **Healthcare that values patient preferences and shared decision making** | Patient preferences for EOL care are discussed and respected | Gjerberg 2015 | Many patients wanted to participate in the decision-making process, although they wanted the staff to make the final decision. Yet, few had been asked about things such as hospitalization or medication when this had been in question. They emphasized that they wanted “their voice to be heard” |
|  |  | Riggs, 2014 | *“If they would just listen to family…sit down and listen…and take into consideration what they say…I lived with him a long time…and I know him better than you…don’t dismiss me, you know?”* |
|  |  | Gray 2020 | Adhering to patient wished  *Many tests and treatments were provided after my mother requested to be left alone. Many of these procedures caused additional pain and disorientation.* |
|  | Different preferences for level of responsibility for decision-making | Gjerberg 2015 | When questioning the patients about who should be involved in such decisions, three categories of answers came up: 1) to participate in the decision-making process, but leave the final decision to the staff, 2) to leave the entire decision making process to the staff, and 3) to have the full responsibility.  Most relatives wanted to be involved in decision making concerning health-care issues if the patient was no longer able to decide on their own, but did not want decision-making authority |
| **Models of care that support quality of life and death with dignity** | Quality of life is highly important in EOL care | Lewis 2019 | a good quality of life is when an individual has control and can meet their own personal standards and expectations. These standards and expectations were perceived as dynamic throughout life and at the EOL as physical and cognitive abilities deteriorate.  Good quality of life was consistently conceptualized as being able to do the things a person enjoys and maintaining their sense of self through these activities, or as the patient’s ability to achieve their aspirations whatever those may be. |
|  | Models of care that support care at home for as long as possible | Devik 2015 | Receiving home nursing care in this situation means to continue a familiar and meaningful life. Having the opportunity to remain in one’s home is highly appreciated. Here, all requisites are known, both inside and outside the house. Memories live in the walls, and in the surrounding nature, and even within  people in the surroundings (family, neighbours, and nurses).  Staying at home is highly valued, and the distance to hospital does not provoke  anxiety. |
|  |  | Agha 2021 | *‘‘They took care of my father as though he was theirs. Staff brought him food from home and local restaurants, kissed and hugged him every chance they got. They treated him with respect and love. I am eternally grateful.’’*  *‘‘They were kind, helpful and they always encouraged him to eat, laugh, have fun and sing songs that uplift people.’’* |
|  |  | Morris, 2020 | *“towards the end the help to manage (her) pain and allow her to stay and die at home was wonderful”* |
|  | Models of care that support quality of life, individuality and death with dignity | Muders, 2015 | A personal farewell is an important aspect, which was also expressed in wishes for more privacy during and after death:  *[My relative] dying in a private room with space for a personal farewell would have been important for me.* |
|  |  | Riggs, 2014 | *“I do think that four people to a room for people who are seriously ill is hard on everybody there…I felt by moving him to a private room that he was allowed to die with some dignity and love and in a peaceful environment”* |
| **Healthcare services that meet patient expectations** | Staff well trained in managing specific conditions and complex issues | Carpenter 2017 | Some surrogates expressed concern about the nursing homes’ ability to manage symptoms. One reported, “They get worse here. That’s why he [sic] sent down to the hospital. Um, most of the time, this is where it starts and then, he gets sent to the hospital. They patch him up a quick fix and send him back here.” |
|  | More comprehensive and responsive services | Muders, 2015 | Faced with many problems, they often felt neglected by time-pressured personnel, particularly by staff at outpatient nursing services:  *The time of nursing service personnel is too limited. Families are overwhelmed with too many problems—for example, constipation, decubitus, edema, shortness of breath, depression, fear, and incontinence.* |
|  | Services that are coordinated and easy to navigate | Lewis 2019 | Both patients and caregivers identified challenges in navigating the health system, although these were conceptualized differently. For patients, attending appointments for care at the EOL could be challenging, with the difficulty of multiple visits to services that were not localized, compounded by poor mobility and the associated costs of transport. |
|  | Availability of accommodation for families at or nearby the medical facilities | Gray 2020 | *They also made sure his family’s needs were met. They made arrangements for us to stay on the hospital grounds without us even asking.* |
|  |  | Agha 2021 | *‘‘Stayed one night with dad and the social workers came the next morning and asked if I stayed here all night? He said they have a facility to stay in and since I had only the clothing on my back the social workers told me they keep new clothing, underwear anything you need. Gave me a Walmart bag and said to go fill it and then they put me up at [Location].’’* |
|  |  | Agha 2021 | ‘‘They never found a room at [Location] or promised hotel, so we never got to say final goodbyes.’’ |
| **Support for family/caregivers in dealing with EOL challenges** | Learning to be a caregiver, including managing symptoms and dealing with communication difficulties | Tarter 2016 | Caregivers described uncertainty about the etiology of pain, difficulties in communicating with patients, lack of consistent guidance from health-care professionals, and secondary suffering as the factors that prevented them from properly and effectively managing pain in their loved ones. |
|  |  | Armstrong 2020 | More education and information about what to expect and how to handle it during EOL care  *I feel like we had a little lack of information as far as meds and how it goes. You know, as the days progress, we’re assuming that her kidneys are shut down and they’re gonna hurt her, so we’re give her more morphine, or—yeah, how does that—how does that go down? I think that would be helpful.* |
|  | Needing to make personal compromises | Lewis 2019 | Caregiver participants described the impact of their loved one’s life-limiting illness as putting their life on hold to care for another, making financial, career and personal compromises in order to do this.  “Juggling” family life and caring responsibilities was a challenge, with many participants highlighting the uneven distribution of caring responsibilities between family members. |
|  | Emotional impacts | Lewis 2019 | Carer participants described EOL care as emotionally difficult. Feelings of guilt, denial, distress and sadness were noted in addition to the physical and logistical challenges of caring for a loved one. |
|  |  | Gray 2020 | Private rooms during the last days of life, expression of staff grief and time with patients immediately after death were highlighted as needed emotional support  *It is my hope and prayer that a more private room situation will one day be available for all Veterans. There were three other patients [in the room]; their families were on a rotating basis during my father’s stay. There was little possibility of privacy of conversation regarding important life or death decisions and intimate health concerns.*  *When he died we were told to take as long staying with him [as we needed]. This was helpful in closure.*  *After his death, his primary (one of them) doctor, Dr. _____ _____ called my sister and I to share his condolences even though he hadn’t been the attending when he died. It was very touching to us, and much appreciated.* |
|  | Support and services for caregivers | Tarter 2016 | Caregivers reported a lack of guidance from healthcare professionals in what to anticipate during the hospice trajectory and a lack of proactive communication especially related to pain. |
|  |  | Agha 2021 | *‘‘When he received his terminal diagnosis, it took way too long for him to be seen by the palliative care doctor and to be adequately managed for pain. Finally, his pain was managed but his emotional distress was not.’’* |

**Supplement 2 Table S2.1 Representation of domain consumer perceptions of quality of care near the end of life (EOL)**

|  | **Study authors /year** | **Domains** | | | | |
| --- | --- | --- | --- | --- | --- | --- |
|  |  | Communication | Healthcare that values patient preferences and shared decision making | Models of care that support quality of life and death with dignity | Healthcare delivery that meets patient expectations | Support for family/caregivers in dealing with EOL challenges |
|  | Agha et al. (2021) | ✓ |  | ✓ | ✓ | ✓ |
|  | Armstrong et al. (2020) | ✓ |  |  |  | ✓ |
|  | Carpenter et al. (2017) | ✓ | ✓ | ✓ | ✓ | ✓ |
|  | Csikai et al. (2010) | ✓ | ✓ |  |  |  |
|  | Devik et al. (2015) | ✓ |  | ✓ | ✓ |  |
|  | Fried et al. 2008) | ✓ | ✓ |  |  |  |
|  | Gjerberg et al. (2015) | ✓ | ✓ | ✓ |  |  |
|  | Gray et al. (2020) | ✓ | ✓ |  | ✓ | ✓ |
|  | Hanson et al. (1997) | ✓ | ✓ | ✓ | ✓ | ✓ |
|  | Krawczyk et al. (2016) | ✓ |  |  |  | ✓ |
|  | Lewis et al. (2019) | ✓ | ✓ | ✓ | ✓ | ✓ |
|  | Low et al. (2014) | ✓ | ✓ | ✓ | ✓ | ✓ |
|  | Morris et al, (2020) | ✓ | ✓ | ✓ | ✓ | ✓ |
|  | Muders et al. (2015) | ✓ | ✓ | ✓ | ✓ | ✓ |
|  | Riggs et al. (2014) | ✓ | ✓ | ✓ | ✓ | ✓ |
|  | Tarter et al. (2016) | ✓ |  |  | ✓ | ✓ |
|  | Waldrop et al. (2012) | ✓ | ✓ |  | ✓ | ✓ |

**Supplement 3, Table S3.1 Characteristics and context of quantitative studies by target population (N=13)**

| **Authors and publication year** | **Country** | **Sample size** | **Target group** | | | **Context** | |
| --- | --- | --- | --- | --- | --- | --- | --- |
|  |  |  | **Patients** | **Relatives** | **Other carers**  **or friends** | **Setting /source of participants** | **Main objective and aspects covered** |
| Richards 2019 | USA | 5435 |  | ✓ |  | (nationwide) Relatives of patients who died in U.S. Department of Veterans Affairs facilities | Bereaved family members’ perceptions of patients’ quality  of care during the last month of life, including  -communication  -support  -pain management |
| Mori 2018 | Japan | 516 |  | ✓ |  | (nationwide) Community sample of family members of patients who had died after receiving palliative care | Bereaved families’  -perception of the need for improvement on the explanations about impending death  -timing of news in relation to time of death  -factors contributing to the need |
| Sanjo 2017 | Japan | 647 |  | ✓ |  | (nationwide) Community sample of bereaved family members of patients who had used palliative care units | Bereaved family members’  -opinion on amount and timing of information provided by oncologist on palliative care for cancer patients at time of first referral  -factors related with perceived adequacy of explanation |
| van der Klink 2010 | NL | 51 |  | ✓ |  | Relatives of patients who had died in a single mixed medical-surgical intensive care unit | -satisfaction with delivery of care  -satisfaction with information provided on sequence of events before death  -patient’s sleep quality  -Need for follow-up bereavement |
| Norris 2007 | USA | 207 |  | ✓ |  | Contacts of decedents (non-sudden deaths) in a single County identified through funeral records | Elements of quality of life for decedents:  -symptom and sickness impact  -pain management rating  -aggressiveness of treatment and health service use  -adherence to care preferences (evidence of family discord)  -life enrichment activities and spiritual care  -communication among patients, family, professional caregivers  -advance care planning & place of death  -satisfaction with care  -community support |
| Teno 2004 | USA | 1,578 |  | ✓ | ✓ | (nationwide) Community sample of bereaved family members or close informants of patients who died of chronic illness | Family centered EOL care/unmet needs:  -whether health workers provided physical comfort and emotional support to patients  -support for decision-making  -treatment with respect  -attended to family’s emotional needs  -provided coordinated care |
| Phipps 2003 | USA | 28 |  | ✓ | ✓ | Bereaved relatives of advanced cancer patients who died at home (supported by healthcare staff) or in institutions | -perception of “how ready” the patient was to die  -satisfaction with care alignment with patient’s wishes (CPR, ventilation and feeding tube)  -emotional support provided by health personnel  -satisfaction with communication from healthcare providers |
| Khandewal 2017 | USA | 2,130 | ✓ | ✓ | ✓ | Nationally representative community sample of Medicare beneficiaries aged >65 years (incl. nursing homes) in last month of life or their families | Patients, relatives or close contacts opinion on whether care was :  -made with family input  -consistent with decedents’ goals of care; or whether decisions would not have been wanted  -Perception of symptom management  -Overall quality end-of-life care (excellent-poor) |
| Parker 2013 | USA | 110 | ✓ | ✓ |  | Patients and their family members receiving palliative care from a large community-based hospice in New England serving urban and suburban populations | Patients’ and family members’ satisfaction with the care they and the patient received |
| Blacquiere 2013 | Canada | 15 |  | ✓ | ✓ | Neurology and neurosurgery units in one institution | Family satisfaction with palliative care received after stroke:  -decision-making  -emotional needs  -artificial feeding / hydration  -communication |
| Heyland 2010 | Canada | 363 | ✓ | ✓ |  | Patients with advanced COPD, CHF, liver disease or cancer from 7 hospitals and their associated home-care programs and their relatives | Satisfaction with and Importance/high priority of:  -spiritual and emotional needs  -relationship with doctors  -communication and decision-making  -illness management |
| Strachan 2009 | Canada | 106 | ✓ |  |  | Patients with advanced heart failure hospitalized in five tertiary teaching hospitals across the country | Opportunities for EOL care improvement and importance of:  -understanding of prognosis  -emotional and physical burden on family  -management: effective symptom relief and place of death  -avoidance of life support if no hope of recovery  -adequate advance plan of care following discharge  -opportunities for honest communication |
| Heyland 2006 | Canada | 440 | ✓ | ✓ |  | Older patients with advanced cancer and chronic end-stage medical diseases in five teaching hospitals across the country | Elements of end-of-life care:  -medical and nursing care  -communication and decision-making  -social relationships and support  -meaningful existence  -advance planning of care |

NL= The Netherlands COPD= Chronic obstructive pulmonary disease CHF= Chronic heart failure

**Supplement 3, Table S3.2 Consumer perspectives on experiences and care quality near the end of life (Quantitative studies N=13 studies), segregated by themes emergent from qualitative studies (see Main Text). See also Summary below (3.1)**

*******Please note that where family reported lack of understanding, we classified this as a deficit in communication.

| **Authors, year, target group** | **Age (yrs)**  **Pt=patients**  **Resp= respondents** | **RR %** | **Communication with patients and family***** | **Patient preference and shared decision making** | **Support for quality of life and death with dignity** | **Patient expectation of staff training, coordination, & responsiveness** | **Emotional Support for caregivers’** |
| --- | --- | --- | --- | --- | --- | --- | --- |
| **BEREAVED RELATIVES** | | | |  |  |  |  |
| Richards 2019 Bereaved relatives  (5,435) | Pt= mean 76  Resp= mean NR | 54.0 | 71% said staff were ‘always’ willing to take time to listen, 67% said staff ‘always’ kept the family informed and 81% said the staff alerted the family before the patient’s death. 80% said staff were kind. caring and respectful. | 77% said staff ‘always’ provided the treatment that patient and family wanted | receipt of palliative care and hospice services were associated with higher overall ratings | 50% said the patient was always/usually not uncomfortable from pain. 63% said personal needs were taken care of. | 59% said staff always provided patient and family spiritual support, and 60% emotional support |
| Mori 2018  Bereaved relatives  (n=516) | Pt= mean 77  Resp= mean 62 | 63.0 | Only 64% reported appropriate timing of being told EOL status, with  10% reporting EOL status ‘never communicated’ |  |  |  | 31% declared need for improvement of clinician explanation on impending death (mostly spouses, relatives of younger, and if low social support) |
| Sanjo 2017  Bereaved relatives  (n=647) | Pt= mean 72  Resp= mean 60 | 71.9 | 11% reported’ no explanation’ about palliative care  Only 40% reported patient & family same information | Only 14% reported Oncologist consulted on desire to know about Rx options | 65% ability to discharge to own home; 45% received information on home-visiting medical services available other than palliative care unit | 11% reported having no one to consult about ‘what to do next’ | 76% had difficulty caring for patient at home;  72% reported Accessible visiting and accommodation for families; 34% reported not knowing when patient can be admitted to palliative care |
| van der Klink 2010  Bereaved relatives  (n=51) | Pt= med 76  Resp= med 53 | 91.0 | 11.8 % reported discrepancy of information provided by ICU staff  13.7% poor staff and family relationships |  |  | 90.2% reported ICU staff very helpful  76.5% felt totally informed by ICU staff  90.2% understood sequence of events | 35.3% needed hospital follow-up bereavement service |
| Norris 2007  Bereaved relatives  (n=207) | Pt= mean 74.1 | 27.0 |  | Only 60.6% of decedents were able (offered opportunity?) to make decisions in last month of life, despite >80% being able to communicate | 82.8% found spiritual involvement helpful- rated as 7+/10.  77.9% found the administration of complementary life-enriching therapies helpful.  50.4% felt patient died at place of choice | 69.4% scored 7+/10 quality of emergency service; 38 - 44% thought staff did not treat most severe symptom in a helpful way; 10-15% felt staff could have done more to control symptom; 18.8% felt patient waited too long for Rx | Having a member of the health care team ensuring the deceased got the best care*; and having a member of the health care team familiar with the deceased who was available nights and weekends** were significant determinants of quality end of life |
| Teno 2004  Bereaved relatives  (n=1,578) | Pt= mean 73.9 | 57.9 | 29.2% had concerns about what to expect while patient dying | 23.9 -30.1% had concerns about physician communication regarding decision-making | 21.1% felt patient not always treated with respect | 15.2% Not enough knowledge of patient for good coordinated care; 22.4 - 50.2% Dissatisfied with support for physical comfort | 49.4% overall satisfied with quality of care;  34.6% had concerns about lack of emotional support to family |
| Phipps 2003  Bereaved relatives  (n=28)  Phipps 2003 (cont) | Pt=mean= 64.0  Resp= NR | 100.0 |  | “The majority” (numeric data not reported) of patients did not want life-sustaining treatments;  No difference (p>0.6) in proportion with living will or POA by place of death | No difference by site of death (p>0.2) in satisfaction with care that ensured death with dignity | No difference (p>0.2) by site of death in care that respected patient wishes (CPR, MV, feeding tube) | Family carers of people who died in institutions were more satisfied (100%) with HCP communication than carers of those who died at home (59% ); No difference in satisfaction with symptom control provided by place of death (p>0.24)  Emotional support in Institutional care more satisfactory (100%) than home care (65%) |
| **PATIENTS/RELATIVES** | |  |  |  |  |  |  |
| Khandewal 2017  Patients or relatives  (n=2,130) | 21% older than 85 | >94.0 | ^a^ Family not kept informed of care plan 29.8% vs. 17%** | ^a^ Decision without enough family input 26% vs. 6.8%**  87.4% reported consistency  with goals of care | ^a^ 23% vs. 12.1 patient not always treated with respect;  ^a^ Unmet need for pain Mx 27.3% vs. 15.2% ** | ^a^ 40% vs 49% reported excellent care ratings  ^a^ 39.8% vs. 44.7% good/very good  ^a^ 20.2% vs. 6.2% fair-poor | ^a^ 45.2% vs 27.4% reported unmet need for addressing sadness/anxiety |
| Parker 2013  Patients or relatives  (n=110) | NR | 21.5 | Median score of 4/4 (very satisfied) for communication related questions like ^“^Availability of the palliative care team to the family” | Median score of 4/4 (very satisfied) for preference/decision making questions like “The way in which the family is included in  treatment and care decisions” | Median score of 4/4 (very satisfied) for QoL questions like “The way in which the palliative care team respects  the dignity of the patient and family” | Median score of 4/4 (very satisfied) for staff competency questions like “How effectively the palliative care team manages the  patient’s (your) symptoms” | Median score of 4/4 (very satisfied) for staff emotional support questions like “Emotional support provided to the patient (you) by the palliative care team” |
| Blacquiere 2013  Patients  (n=15) | Pt= med 86 | 55.0 | Communication with patient score 9.2/10; Higher dissatisfaction with: communication with family | Higher satisfaction with participation in decision-making than with coordination of care and attention to emotional needs | Ensuring dignified death score 9.1 /10  Respectful medical care 9.15/10 | High dissatisfaction with feeding & hydration | Overall satisfaction score 9.04 out of 10; Emotional support for families 8.8/10 |
| Heyland 2010  Patients & relatives  (n=363) | Pt= mean 76.6  Resp= mean 61.9 | 77.1 |  | Room for improvement: decision-making a priority | Preserving dignity, while being cared for,  & being treated with respect and compassion were associated with perceived good quality EOL care | 9 - 57% of patients completely satisfied; Room for improvement: highest in priority was emotional support provided to patients | Global family satisfaction on how they were treated 4.2 out of 5 |
| Strachan 2009  Strachan (cont)  Patients (n=106) | Pt= mean 75 | NR | 51.5% reported Extremely important to have honest discussion with doctor but 29.7% satisfied;  23.8% valued opportunity to discuss fears of dying | 24.3% think it’s extremely important to be involved in decisions about care they receive, but only 19.6% fully satisfied | 24% satisfied with being treated as individual with values; 25.7% satisfied with preserving dignity; 28.3% satisfied with respectful and compassionate care;  58.5% felt burden on family would prevent home death;  13.3% extremely important to die at place of choice | 61% rated it extremely important to avoid life support if no hope of recovery | 48.1% thought lack of health services would be obstacles for home death; 38.6% important to have home care plan on discharge but only 18% satisfied about it. |
| Heyland 2006  Patients  (n=440) | Pt= mean 71.2  Resp= mean 56.5 | 77.0 | Reported the following to be Extremely important:  To have honest communication from doctor  P=44.1% R=72%  To have opportunity to discuss fears of death:  P= 24.7% R=31.3% | Reported the following to be Extremely important: To be involved in decisions about care received: P=28.1% R=54.4%  To receive help about difficult Rx decisions: P=14.7% R=33.8%  Not to be kept alive on life support if no hope of recovery:  P=55.7% R=73.2% | Reported the following to be Extremely important:  To receive Rx that preserves dignity P=27.3%  To prepare for life’s end; P=43.9% R=66%  To receive respectful and compassionate care: P=31.6% R=47.5%  Not to be a burden to family: P=41.8%  To have patient die at place of choice: P=17.1% R=50% | Reported the following to be Extremely important:  To receive adequate symptom relief  P=38.9% R= 70.6%  Extremely important to have trust and confidence in your doctors  P=55.8% R=75.3% | Reported the following to be Extremely important to have a home plan of care  P=41.8% R= 71.6% |

** p< 0.05 ** p<0.01 RR%= Response rate % CPR= cardiopulmonary resuscitation EOL= end-of-life HCP= Healthcare provider*

*MV= mechanical ventilation Mx=Management NR= Not reported POA=Power of Attorney Pt=patient perspective*

*Resp=other respondent/family perspective RR%=response rate% Rx= Treatment*

***^a^****= rating when care was inconsistent with goals of care vs. when care was consistent with goals of care*

**Supplement 3,** **Summary 3.1. Detailed findings from the quantitative studies (13 surveys)**

Three studies of bereaved relatives [2-4] and four of patients [5-8] addressed all five domains identified in the qualitative framework; the remaining 6 studies supported various aspects of the framework.

**Communication**

While honest communication around death was seen as important [5, 7], communication failures were highlighted in 9/11 surveys, with the main issues being contradictory information given to patients and families [3, 9], poor timing or lateness of news disclosure [10], limited or no explanations given on palliative care [3], or lack of information on the loved one’s care plan [11]. By contrast, communication with healthcare providers was also highly rated by others [4, 8], and seen as an opportunity to discuss fears of death [5, 7], and for families to receive emotional support [6]. Some bereaved relatives reported higher satisfaction with end-of-life communication in hospitals and hospices than among those dying at home [12].

**Healthcare which values patient preferences and shared decision making**

Preferences of care were also examined, with patients in two surveys [5, 7], and bereaved relatives in another [12], emphasising the importance of avoiding futile life support. Three studies involving families consistently reported high family involvement and alignment of patients wishes and actual treatment [4, 8, 11], and this did not vary by place of death [12]. Symptom control was perceived to be suboptimal by at least one in five bereaved relatives [2, 11, 13].

Despite the importance of shared decision-making being acknowledged by patients [5, 7], surveys of relatives pointed out room for improvement [14]. Deficits were identified in enabling autonomous decision in the last month of life [13], (dis)satisfaction with decisions made without enough family input [11], and clinician communication for decision-making [2]. For other patients, satisfaction with decision-making was higher than satisfaction with care coordination [6].Interestingly, prevalence of advance care documentation did not vary by place of death [12].

Looking at the theme of death with dignity, the most salient feature on patient surveys was a perception of respectful and compassionate care as important features of good-quality service [5-7, 14]. In two surveys of relatives, family members reported not always feeling treated with respect [2, 11]. Receipt of hospice care was associated with higher quality ratings [4]. A survey of bereaved relatives found no difference in satisfaction with care achieving death with dignity, whether people died at home or in an institution [12].

In relation to patient expectations of skilled staff, the importance of clinicians treating symptoms optimally to support physical comfort was mentioned by both relatives [2, 4, 13], and patients [5, 8].

Regarding system coordination and responsiveness, while some were satisfied with the team effectiveness [4, 8], the main difficulties navigating the health system were in relation to responsibilities and access to support for those wanting to be cared at home. Patients considered a home plan of care [5, 7], and confidence in their own doctors [5] very important , but at the same time there was awareness of the gaps, both in staff knowledge of patients [2], and in availability to families, to be consulted about next steps [3].

Finally, patients and caregivers highlighted the need for staff to support relatives in the difficulties they face caring for dying patients at home [3, 13] [7], with such difficulties including not knowing when the right time was for palliative care admission, lacking community services that would allow a home death, and the need for follow-up bereavement counselling [3, 7]. Somewhat reassuringly, emotional and spiritual support drew good satisfaction scores (59% to >70%) from staff to families [4, 8] when care was provided in institutions [3, 12].

**REFERENCES FOR QUANTITATIVE STUDIES**

1. Cardona M, Lewis ET, Kristensen MR, Skjot-Arkil H, Ekmann AA, Nygaard HH, Jensen JJ, Jensen RO, Pedersen JL, Turner RM *et al*: **Predictive validity of the CriSTAL tool for short-term mortality in older people presenting at Emergency Departments: a prospective study**. *European geriatric medicine* 2018, **9**(6):891-901.

2. Teno JM, Clarridge BR, Casey V, Welch LC, Wetle T, Shield R, Mor V: **Family perspectives on end-of-life care at the last place of care**. *Jama* 2004, **291**(1):88-93.

3. Sanjo M, Morita T, Miyashita M, Sato K, Kamibeppu K, Tsuneto S, Shima Y: **Are Bereaved Family Members Satisfied With Information Provision About Palliative Care Units in Japan?** *Am J Hosp Palliat Care* 2018, **35**(2):275-283.

4. Richards CA, Liu CF, Hebert PL, Ersek M, Wachterman MW, Reinke LF, Taylor LL, O'Hare AM: **Family Perceptions of Quality of End-of-Life Care for Veterans with Advanced CKD**. *Clin J Am Soc Nephrol* 2019, **14**(9):1324-1335.

5. Heyland DK, Dodek P, Rocker G, Groll D, Gafni A, Pichora D, Shortt S, Tranmer J, Lazar N, Kutsogiannis J *et al*: **What matters most in end-of-life care: perceptions of seriously ill patients and their family members**. *Cmaj* 2006, **174**(5):627-633.

6. Blacquiere D, Bhimji K, Meggison H, Sinclair J, Sharma M: **Satisfaction with palliative care after stroke: a prospective cohort study**. *Stroke* 2013, **44**(9):2617-2619.

7. Strachan PH, Ross H, Rocker GM, Dodek PM, Heyl, DK: **Mind the gap: Opportunities for improving end-of-life care for patients with advanced heart failure**. *Can J Cardiol* 2009

**25**(11):635-640.

8. Parker SM, Remington R, Nannini A, Cifuentes M: **Patient Outcomes and Satisfaction With Care Following Palliative Care Consultation**. *Journal of Hospice & Palliative Nursing* 2013, **15**(4):225-232.

9. van der Klink MA, Heijboer L, Hofhuis JG, Hovingh A, Rommes JH, Westerman MJ, Spronk PE: **Survey into bereavement of family members of patients who died in the intensive care unit**. *Intensive Crit Care Nurs* 2010, **26**(4):215-225.

10. Mori M, Morita T, Igarashi N, Shima Y, Miyashita M: **Communication about the impending death of patients with cancer to the family: a nationwide survey**. *BMJ Support Palliat Care* 2018, **8**(2):221-228.

11. Khandelwal N, Curtis JR, Freedman VA, Kasper JD, Gozalo P, Engelberg RA, Teno JM: **How Often Is End-of-Life Care in the United States Inconsistent with Patients' Goals of Care?** *J Palliat Med* 2017, **20**(12):1400-1404.

12. Phipps E, Braitman L: **Family caregiver satisfaction with care at end of life: Report from the cultural variations study (CVAS)**. *American Journal of Hospice and Palliative Medicine* 2004, **21**:340 - 342.

13. Norris K, Merriman MP, Curtis JR, Asp C, Tuholske L, Byock IR: **Next of kin perspectives on the experience of end-of-life care in a community setting**. *J Palliat Med* 2007, **10**(5):1101-1115.

14. Heyland DK, Cook DJ, Rocker GM, Dodek PM, Kutsogiannis DJ, Skrobik Y, Jiang X, Day AG, Cohen SR, Canadian Researchers at the End of Life N: **Defining priorities for improving end-of-life care in Canada**. *CMAJ* 2010, **182**(16):E747-752.
